# Mg^2+^-Dependent Control of the Spatial Arrangement of *Rhodococcus erythropolis* PR4 Cells in Aqueous-Alkane Two Phase Culture Containing *n*-Dodecane

**DOI:** 10.1264/jsme2.ME15196

**Published:** 2016-05-14

**Authors:** Hayato Takihara, Yumiko Akase, Michio Sunairi, Noriyuki Iwabuchi

**Affiliations:** 1 Laboratory of Molecular Microbiology, Department of Applied Biological Science, College of Bioresource Sciences, Nihon University1866 Kameino, Fujisawa, Kanagawa 252–8510Japan

**Keywords:** organic solvent tolerance, *Rhodococcus*, alkane, cell translocation, spatial arrangement of cells

## Abstract

We recently reported that a close relationship exists between alkane carbon-chain length, cell growth, and translocation frequency in *Rhodococcus*. In the present study, we examined the regulation of the spatial arrangement of cells in aqueous-alkane two phase cultures. An analysis of the effects of minerals on cell localization revealed that changes in the concentration of MgSO_4_ in two phase cultures containing *n*-dodecane (C12) altered cell localization from translocation to adhesion and vice versa. Our results indicate that the spatial arrangement of cells in two phase culture systems is controlled through the regulation of MgSO_4_ concentrations.

The productive use of living bacteria in white biotechnology and bioremediation applications requires a thorough understanding of the interactions between bacterial cells and organic solvents. White biotechnology is the industrial application of biotechnology processes to the synthesis of products that are easily degradable, require less energy to produce, and create less waste during their production (2). Rhodococci are promising host strains for use in white biotechnology applications because they have the ability to degrade a number of aliphatic and aromatic hydrocarbons, such as PCBs (1). Aqueous-organic solvent two phase culture systems exploit living bacteria to produce goods for industrial and/or medical use from lipophilic substances and are widely used in white biotechnology. In order to maximize the efficiency of aqueous-alkane two phase culture methods for use in white biotechnology, the spatial arrangement between the substrate and host cell must first be optimized (3).

Another important factor in aqueous-alkane two phase culture systems is the tolerance of the host strain to organic solvents. Various engineering techniques have been used to increase the tolerance of host cells to toxic organic solvents while maintaining their biotechnologically useful activity. More efficient white biotechnology methods may be developed by combining strategies to enhance bacterial tolerance to organic solvents with strategies to regulate the spatial arrangement between the substrate of interest and cells in culture.

We previously reported that *Rhodococcus erythropolis* PR4 cells in a two phase culture completely translocated to and then multiplied in the pristane (C19) phase and also that a close relationship existed between growth, cellular localization, and alkane carbon-chain length (4). In addition, we recently reported that a close relationship exists between alkane carbon-chain length, growth, and the frequency of translocation to the alkane phase in various *Rhodococcus* strains (6). These findings suggest that many *Rhodococcus* strains have the ability to translocate to and subsequently grow in alkane phases composed of solvents with longer carbon chains; however, the degrees of translocation and growth vary depending on the alkane tolerance of different strains.

In our previous studies examining the alkane tolerance of rhodococci, we demonstrated that many *Rhodococcus* strains are capable of changing their localization in two phase cultures depending on the length of the alkane chain (6). Moreover, the up-regulation/overexpression of GroEL2 has been shown to alter the localization behavior of some *Rhodococcus* strains (5, 6). These findings prompted us to hypothesize that the activity of *Rhodococcus* cells in aqueous-alkane two phase culture systems may be optimized by regulating the spatial arrangement between cells and the alkane phase. Therefore, we herein examined the factors influencing the localization of rhodococci in aqueous-alkane two phase cultures.

Since IB2 broth (Glucose 10 g, Bacto-Yeast extract [Difco] 10 g, [NH_4_]_2_SO_4_, 0.5 g; NaCl, 0.1 g; MgCl_2_·6H_2_O, 0.18 g; CaCl_2_, 0.132 g; FeCl_2_·6H_2_O, 0.01 g; L^−1^, pH 7.2) consists of glucose, yeast extract, and minerals, we first examined the effects of glucose and yeast extract on bacterial localization. At the concentrations examined, neither glucose nor yeast extract had any effect on the localization of *R. erythropolis* PR4 cells. Regardless of the glucose or yeast extract concentration, PR4 cells translocated in the presence of C19 and exhibited adhesion in the presence of C12 (data not shown).

MM medium ([NH_4_]_2_SO_4_, 0.5 g; NaCl, 0.1 g; MgCl_2_·6H_2_O, 0.18 g; CaCl_2_, 0.132 g; K_2_HPO_4_, 0.5 g; FeCl_2_·6H_2_O, 0.01 g; L^−1^, pH 7.2) was used to examine the effects of various minerals on bacterial localization. An aliquot of a washed cell suspension derived from a stationary-phase culture was transferred to fresh MM medium amended with C12 or C19 (5%). The initial cell density was 10^6^ colony forming units mL^−1^, and the culture was incubated at 28°C for 5 d with shaking (110 rpm). In our previous two phase culture experiments based on IB2 medium, cell localization was categorized based on which phase the majority of cells were located within (4). Cells were categorized as having adhered (ADH) at the aqueous-alkane phase interface or translocated (TRN) into the alkane phase (Fig. S1). When PR4 cells were cultured in MM medium amended with C19, PR4 cells clearly translocated to the C19 phase. On the other hand, images taken during the course of two phase culture experiments based on MM medium amended with C12 showed variable results. For example, a small number of TRN cells were observed in the C12 droplets surrounded by numerous ADH cells. Henceforth, we categorized localization based on the following strategy.

When ADH and TRN cells were observed in the same droplet, localization was determined based on the type of localization behavior exhibited by the majority of cells in the droplet. For example, if the number of ADH cells exceeded the number of TRN cells in the same droplet, we categorized the sample as ADH>TRN. If the opposite was observed, we categorized the sample as TRN>ADH. This localization categorization (ADH>TRN or TRN>ADH) was used for each photograph. In cases in which the type of cell localization in a photograph was clearly discerned, we categorized the localization as either ADH or TRN, as described above. At least 5 independent experiments were performed, and 5 photographs were taken for each condition (*n*=25). The percentage of ADH, TRN, ADH>TRN, or TRN>ADH cells in each category was calculated for each condition examined.

The effects of minerals on cell localization were examined as described above. When PR4 cells were cultured in MM medium amended with C19, 100% of the photographs were categorized as TRN (Table S1). On the other hand, 65% of the photographs used to determine localization were categorized as ADH when PR4 cells were cultured in MM medium amended with C12. These results were basically consistent with our previous findings (4).

In order to examine the effects of each mineral contained in IB2 medium on PR4 translocation separately, cells were cultured in NP medium (K_2_HPO_4_, 0.5 g; [NH_4_]_2_SO_4_, 0.5 g; L^−1^, pH 7.2) amended with 5% of C19 or C12 (v/v) containing the respective mineral of interest. Regardless of the minerals present, 100% of the photographs were categorized as TRN when C19 was added to medium (Table S1). A total of 62% of the photographs used to determine translocation were categorized as TRN (Table S2), indicating that PR4 cells primarily translocated to the C12 phase in NP medium. A typical photograph is shown in Fig. 1A. These results showed that PR4 cells are essentially able to translocate to C12 and that some of the minerals in MM broth inhibit this translocation. On the other hand, the minerals examined in this test had no effect on the localization behavior of PR4 cells when C19 was added to NP medium (Table S1). Based on these results, we focused on the effects of the minerals on changes in the localization behavior of PR4 cells in NP medium amended with C12 in downstream analyses.

In an attempt to confirm this result, we also examined the effects of FeCl_2_, CaCl_2_, MgCl_2_, and NaCl on the localization behavior of PR4 cells in a two phase culture in the presence of C12, and the results obtained are summarized in Table S2. Approximately 60% of the photographs used to determine localization were categorized as ADH or ADH>TRN when MgCl_2_ was added to NP medium at the same concentration as that in MM medium (0.18 g L^−1^), indicating that cells primarily localized at the aqueous-C12 interface (Table S2 and Fig. 1B) in the presence of MgCl_2_. In contrast, more than 60% of the photographs used to determine localization were categorized as TRN in NP medium containing the other minerals examined, indicating that the presence of these minerals did not significantly affect the localization behavior of PR4 cells (Table S2). These results suggest that MgCl_2_ inhibits the translocation of PR4 cells to the C12 phase in a two phase culture with NP medium.

We then investigated the effects of MgCl_2_ concentrations on the translocation of PR4 cells to the C12 phase (Table S2). At a low (0.09 μM) MgCl_2_ concentration, 88% of the photographs used to determine localization were categorized as TRN. In contrast, at MgCl_2_ concentrations of 0.9 μM and greater, the photographs were evenly distributed between the various categories (16–40%), with one exception of 0.9 μM-ADH (4%). These results suggest that MgCl_2_ exerts moderate concentration-dependent effects on cell localization in NP medium. A threshold between adhesion and translocation was observed between 0.9 and 0.09 μM MgCl_2_. Hence, the minimum concentration of MgCl_2_ needed to inhibit the translocation of PR4 cells to the C12 phase was estimated to be 0.9 μM, and subsequent experiments were carried out in medium with 0.9 μM Mg^2+^.

In order to determine whether inhibiting the translocation of PR4 cells to the C12 phase is limited to MgCl_2_ only, the effects of other magnesium-containing compounds were examined. The concentration of Mg^2+^ was adjusted to 0.9 μM for each compound. Typical photographs are shown in Fig. 1B (MgCl_2_) and Fig. S2 (Mg[CH_3_COO]_2_, Mg[NO_3_]_2_, Mg[CH_3_-CH[OH]-COO]_2_, or MgSO_4_), and photograph categorizations are listed in Table S2. Most of the photographs used to determine translocation were categorized as Adh in medium containing Mg(CH_3_-CH[OH]-COO)_2_, Mg(NO_3_)_2_, or MgSO_4_, indicating that PR4 cells localize at the aqueous-C12 interface under these conditions. In NP medium containing Mg(CH_3_COO)_2_, 60% of the photographs were categorized as either ADH or ADH>TRN. Although this value was lower than that observed under the other conditions examined, we judged cell localization to be ADH. These results suggest that Mg^2+^ inhibits the translocation of PR4 cells in two phase cultures based on NP medium and C12 when present at a concentration greater than 0.9 μM.

In the experiments described above, magnesium-containing compounds were added to medium before cultivation; therefore, we subsequently investigated the effects of adding Mg^2+^ during cultivation on the translocation of PR4 cells to the C12 phase. PR4 cells were initially grown in a two phase culture with NP medium and C12, and a check of the localization behavior revealed that the cells had translocated. Magnesium-containing compounds were then added to the aqueous phase at final concentrations of 0.9 mM. The samples were cultured for an additional 3 d, after which the cell localization behavior was categorized (Table S3 and Fig. S3).

When Mg(CH_3_COO)_2_, Mg(CH_3_-CH[OH]-COO)_2_, Mg(NO_3_)_2_, or MgCl_2_ was added to the aqueous phase during cultivation, more than 84% of the photographs were categorized as TRN or TRN>ADH (Table S3). In contrast, when MgSO_4_ was added to the aqueous phase during cultivation, PR4 cells localized at the surface of the C12 phase. These results indicate that MgSO_4_ inhibits the localization of PR4 cells to the C12 phase when added during cultivation.

Dose-response experiments were performed to obtain a better understanding of the inhibitory effects of MgSO_4_ on the translocation of PR4 cells (Table S3 and Fig. 2). When MgSO_4_ was added to the aqueous phase in the two phase culture at less than its estimated minimum inhibitory concentration, cells translocated, as previously observed (Fig. 2E and F). In contrast, cells adhered to the C12 phase when MgSO_4_ was added to the aqueous phase at a final concentration of 9.0 μM (Fig. 2D). Under this condition, 69% of the photographs were categorized as ADH or ADH>TRN (Table S3), indicating that translocation to the C12 phase is inhibited when MgSO_4_ is added to the aqueous phase at concentrations of 9.0 μM of higher during cultivation. This Mg^2+^ concentration was 10-fold higher than that used in experiments in which Mg^2+^ was added before cultivation, suggesting that Mg^2+^ has a greater influence on cells within the aqueous phase than on cells localized at the aqueous-alkane interface. Therefore, the estimated minimum inhibitory concentration of Mg^2+^ added during cultivation was higher than when it was added before cultivation.

In order to assess the effects of SO_4_^2−^ on the observed change in cell localization, CaSO_4_, Na_2_SO_4_, K_2_SO_4_, or (NH_4_)_2_SO_4_ was added to the aqueous phase in the two phase culture during cultivation at a final concentration of 0.9 mM, and cell localization was determined after 3 d. No significant changes were observed in cell localization behavior from translocation to adhesion under any of the conditions examined (Table S3). These results indicate that Mg^2+^ derived from MgSO_4_ is the essential component for altering cell localization behavior from translocation to adhesion during cultivation in two phase cultures containing C12.

As described above, we evaluated the timing of the addition of MgSO_4_, the concentration of MgSO_4_ in the aqueous phase as well as the effects of different magnesium-containing compounds on the localization behavior of PR4 cells in a two phase culture with C12 alkane. The results obtained revealed that MgSO_4_ significantly affected the localization behavior of PR4 cells during cultivation. Therefore, we attempted to develop a one-round culture protocol using MgSO_4_ in order to control the spatial arrangement of PR4 cells in two phase culture systems based on NP medium with C12. Our results are illustrated in Fig. 3, the relevant categorizations are summarized in Table S4, and the method was described in supplemental Materials and Methods.

In step 1, PR4 cells were cultured in NP+MgSO_4_ (0.9 μM) medium in the presence of C12 alkane, and a check of localization indicated that the cells had adhered, indicating that 100% of the photographs were categorized as ADH. In step 2, 9 mL of the aqueous phase was pipetted carefully and 10 mL of fresh NP medium was added to the same tube, thereby reducing the MgSO_4_ concentration in the aqueous phase to 0.09 μM. The sample was cultured for 3 d, at which time the cells were found to have translocated (100%). In step 3, MgSO_4_ was added to the culture sample to a final concentration of greater than 9.0 μM. The sample was cultured for 3 d, after which the localization behavior was examined and categorized as ADH (100%). In step 4, glucose solution and *n*-octane (C8) were added to the aqueous and C12 phases, respectively, as described in the Supplemental Materials, in order to induce the release of adherent cells from the aqueous-C12 interface to the aqueous phase. After 3 d of cultivation, the aqueous phase was sampled, and the number of cells in the aqueous phase was determined by a most probable number (MPN) analysis because it was assumed that the detection of cells released from the aqueous-C12 interface to the aqueous phase by a plating method may be very difficult.

When glucose solution and C8 were added to the two phase culture, the number of viable cells was 5.6×10^2^ cells mL^−1^, whereas viable cells were not detected in the aqueous phase in cultures in which no glucose solution or C8 were added or before the addition of glucose solution or C8. Before this experiment, our working hypothesis with respect to step 4 was that the release of cells from the interface to the aqueous phase was likely caused by the addition of C8 alkane (which is more toxic than C12) and glucose solution (as a nutrient source). As expected, the detection of viable cells by the MPN analysis and the observation of cells in a sample of the aqueous phase examined using phase-contrast microscopy (Fig. S4) revealed that some of the cells localized at the aqueous-alkane interface may have been released to the aqueous phase. To the best of our knowledge, this is the first reported observation regarding the localization of PR4 cells in the aqueous phase after cultivation in aqueous-alkane two phase cultures, and the results obtained are significant for controlling the spatial arrangement of PR4 cells.

In the present study, we found that MgSO_4_ plays an important role in regulating the localization of PR4 cells in two phase cultures containing C12. By varying the point at which MgSO_4_ is added to the culture, the concentration of MgSO_4_ in the aqueous phase during the cultivation, and the type of magnesium-containing compound added to the two phase culture, we developed a culture strategy to control the spatial arrangement of PR4 cells during a one-round cultivation. The exposure of bacteria to toxic substrates and products is considered to be a major issue in whole-cell biotransformation processes using two phase culture systems (3). However, the two phase culture strategy presented here may minimize the exposure of bacteria to toxic compounds. By combining strategies to regulate the spatial arrangement between substrates and cells of interest with strategies to enhance bacterial tolerance to organic solvents, more efficient whole-cell biotransformation methods may be developed for use in white biotechnology.

It currently remains unclear (i) why the presence of magnesium-containing compounds in the two phase culture system inhibits translocation to the C12 phase; (ii) why only Mg^2+^ derived from MgSO_4_ alters the localization behavior of PR4 cells from TRN to ADH during cultivation; (iii) how Mg^2+^ derived from MgSO_4_ or other magnesium-containing compounds affects the surface properties of PR4 cells, resulting in a change in Gibbs energy; and (iv) how PR4 cells respond overall to the addition of MgSO_4_ and/or other magnesium-containing compounds in two phase culture systems. In an attempt to clarify these questions, we have begun a proteogenomic/physicochemical study (data will appear elsewhere). Although further studies are needed, our results will contribute to efforts to develop more efficient white biotechnology strategies utilizing living bacteria.

## Supplementary Material



## Figures and Tables

**Fig. 1:**
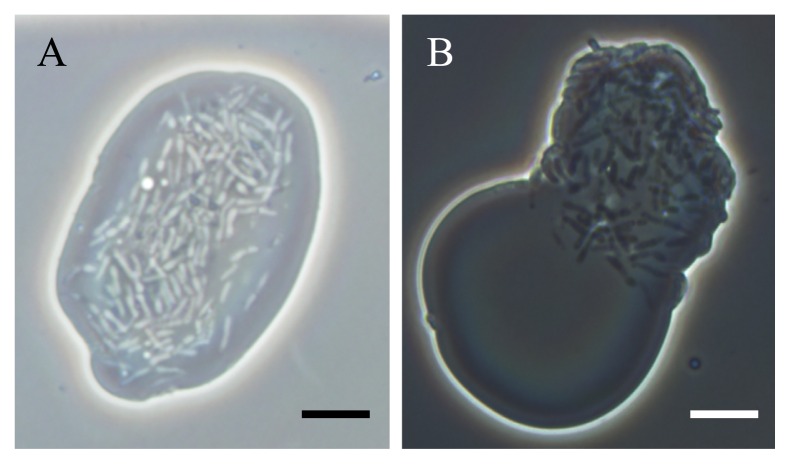
Phase-contrast micrographs illustrating the localization of *R. erythropolis* PR4 cells grown in NP medium (A) or NP+MgCl_2_ (B) containing C12 alkane. MgCl_2_ was added to medium at the same concentration as that in MM medium (0.18 g L^−1^). Both photographs were taken at the same magnification. Scale bar=5 μm.

**Fig. 2:**
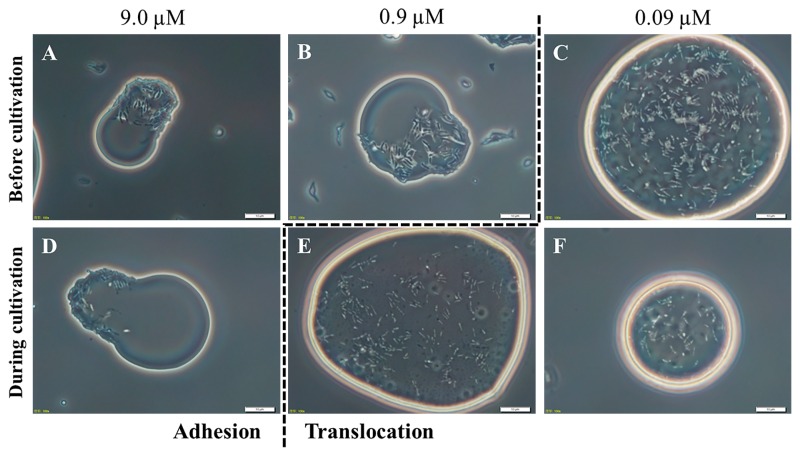
Effects of the addition of MgSO_4_ during cultivation on the localization of PR4 cells in a two phase culture containing C12 alkane. In photographs A, B, and C, MgSO_4_ was added to medium before cultivation, and the sample was cultured for 5 d. In photographs D, E, and F, MgSO_4_ was added to medium after 3 d of cultivation, and the sample was cultured for an additional 3 d. The concentrations of MgSO_4_ in the samples represented by photographs A and D, B and E, and C and F were 9.0, 0.9, and 0.09 μM, respectively. The dashed line indicates the threshold between adhesion and translocation. All photographs were taken at the same magnification. Scale bar=10 μm.

**Fig. 3:**
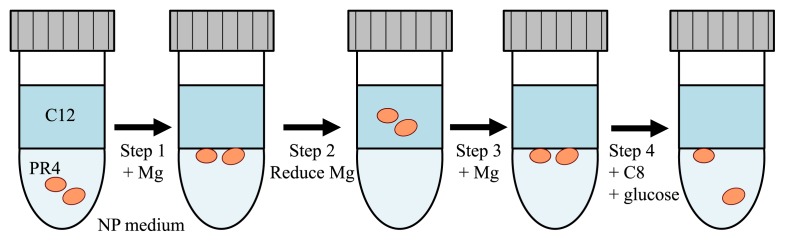
Illustration of the Mg^2+^-dependent control of the spatial arrangement of PR4 cells in a two phase culture in the presence of C12.
