# Supplementary material for: Mg2+-Dependent Control of the Spatial Arrangement of Rhodococcus erythropolis PR4 Cells in Aqueous-Alkane Two Phase Culture Containing n-Dodecane
Source: Microbes Environ. 2016 May 14;31(2):178–81. doi: 10.1264/jsme2.ME15196 (PMC4912155; doi:10.1264/jsme2.ME15196)

Supplemental materials

Mg-dependent control of the spatial arrangement of *Rhodococcus erythropolis* PR4 cells in aqueous-alkane two phase culture containing *n*-dodecane

Hayato Takihara, Yumiko Akase, Michio Sunairi, Noriyuki Iwabuchi*.

[1] Laboratory of Molecular Microbiology, Department of Applied Biological Science, College of Bioresource Sciences, Nihon University, 1866 Kameino, Fujisawa, Kanagawa 252-8510, Japan.

Running title: Mg-dependent control of cell localization

*Corresponding author: Noriyuki Iwabuchi
Mailing address: Laboratory of Molecular Microbiology, Department of Applied Biological Science, College of Bioresource Sciences, Nihon University, 1866 Kameino, Fujisawa, Kanagawa 252-0880, Japan
Tel: +81-466-84-3354; Fax: +81-466-84-3354
E-mail: iwabuchi@brs.nihon-u.ac.jp.

Supplemental materials and methods

Inducing the release of adherent cells from the aqueous-alkane phase interface to the aqueous phase

A 10-ml volume of NP medium was prepared for this experiment, and 0.5 ml of C12 was added to the medium at a concentration of 5%. Pre-cultured and washed *R. erythropolis* PR4 cells were inoculated into an aqueous phase of the NP medium containing C12 at an initial cell density of 10^6 colony forming units per ml.

In step 1, $MgSO_4$ was added an aqueous phase of the NP medium containing C12 at a final concentration of 0.9 μM, and resulted sample was cultured for 3-5 days at 28°C with shaking (110 rpm). The localization behavior of the cells was then assessed, and it was found that almost all of the cells were localized at the interface of the aqueous and C12 phases.

In step 2, 9 ml of the aqueous phase was pipetted carefully and 10 ml of fresh NP medium was added to an aqueous phase of the same tube, thus reducing the $MgSO_4$ concentration in the aqueous phase to 0.09 μM. The sample was cultured for 3 days, at which time localization behavior of the cells was then assessed, and it was found that almost all of the cells were localized in C12 phase.

In step 3, $MgSO_4$ was added to an aqueous phase of the culture sample to a final concentration of greater than 9.0 μM. The sample was cultured for 3 days, at which time localization behavior of the cells was then assessed, and it was found that almost all of the cells were localized at the interface of the aqueous and C12 phases.

In step 4, glucose solution was added to the aqueous phase at a final concentration of 1%, and then 0.5 ml of C8 was added to the C12 phase. The sample was then cultured for an additional 3-5 days. After cultivation, the sample was transferred to a

sterilized, stoppered syringe and allowed to stand at room temperature until the aqueous and C12 phases had completely separated. The aqueous phase (lower phase) was carefully collected and transferred to a new tube without disturbing the alkane phase. Next, 1 ml of the collected sample was added to 9 ml of IB2 broth, and serial 10-fold dilutions of the cell suspension were subsequently prepared using IB2 broth. These samples were cultivated for 2 days at 28°C with shaking (110 rpm), and the number of viable cells was determined by most probable number (MPN) analysis, using an increase in turbidity of the IB2 medium as an indicator of cell growth. An aliquot of the sample was also observed by phase-contrast microscopy (Fig. S4).

Supplemental Tables

Table S1, Takihara *et al.*

Table S1. Categorization of photographs used for determining the localization of *R. erythropolis* PR4 cells cultivated in a two phase system with C19.

Conditions	Cell localizations				n=
	Adhesion	ADH > TRN	TRN > ADH	Translocation	
MM medium	0	0	0	100	25
NP medium	0	0	0	100	25
NP medium +					
$CaCl_2$ (0.7 mM)	0	0	0	100	25
$FeCl_2$ (0.1 mM)	0	0	0	100	25
NaCl (1.7 mM)	0	0	0	100	25
$MgCl_2$ (0.9 mM)	0	0	0	100	25

Five independent experiments were performed, and 5 photographs were taken for each condition (n=25). The percentage of photographs categorized as ADH, TRN, ADH > TRN, or TRN > ADH was calculated for each condition examined. When the effects of $FeCl_2$, $CaCl_2$, $MgCl_2$, and NaCl were examined separately, each mineral was added to NP medium at the same concentration as in MM medium.

Table S2. Categorization of photographs used for determining the localization of *R. erythropolis* PR4 cells cultivated in a two phase system with C12 in which magnesium-containing compounds were added before cultivation.

Conditions	Cell localizations				n=
	Adhesion	ADH > TRN	TRN > ADH	Translocation	
MM medium	65	0	12	23	66
NP medium	25	5	8	62	63
NP medium +					
$CaCl_2$ (0.7 mM)	0	16	19	65	57
$FeCl_2$ (0.1 mM)	0	5	29	65	38
NaCl (1.7 mM)	15	0	24	62	34
$MgCl_2$ (0.9 m M)	33	26	16	25	76
$CaCl_2$, $FeCl_2$ and NaCl	4	4	23	69	26
$MgCl_2$ concentration (μM)					
45.2	24	32	16	28	25
9	20	24	24	32	25
0.9	4	40	20	36	25
0.09	0	0	12	88	25
Magnesium compounds					
$Mg(CH_3COO)_2$	57	3	26	14	35
$Mg(CH_3\text{-}CH(OH)\text{-}COO)_2$	100	0	0	0	45
$Mg(NO_3)_2$	100	0	0	0	29
$MgSO_4$	92	3	5	0	39

At least 5 independent experiments were performed, and 5 photographs were taken for each condition (n>25). The percentage of photographs categorized as ADH, TRN, ADH > TRN, or TRN > ADH was calculated for each condition examined. When the effects of $FeCl_2$, $CaCl_2$, $MgCl_2$, and NaCl were examined separately, each mineral was added to NP medium at the same concentration as in MM medium. When the effects of

magnesium compounds were examined separately, the Mg^{2+} concentration was adjusted to 0.9 μM for each compound.

Table S3. Categorization of photographs used for determining the localization of *R. erythropolis* PR4 cells cultivated in a two phase system with C12 in which magnesium-containing compounds were added during cultivation.

Conditions	Cell localizations				n=
	Adhesion	ADH > TRN	TRN > ADH	Translocation	
Magnesium compounds					
$Mg(CH_3COO)_2$	0	0	17	83	36
$Mg(CH_3\text{-}CH(OH)\text{-}COO)_2$	5	11	42	42	38
$Mg(NO_3)_2$	0	3	18	79	33
$MgSO_4$	100	0	0	0	46
$MgCl_2$	1	1	15	83	80
$MgSO_4$ concentration (μM)					
9.0	66	3	16	16	38
0.9	3	16	13	67	32
0.09	0	0	0	100	29
Sulfates					
$CaSO_4$	0	0	7	93	29
Na_2SO_4	0	12	19	69	42
K_2SO_4	0	0	0	100	22
$(NH_4)_2SO_4$	0	0	44	56	34

At least 5 independent experiments were performed, and 5 photographs were taken for each condition (n>25), except for K_2SO_4. The percentage of photographs categorized as ADH, TRN, ADH > TRN, or TRN > ADH was calculated for each condition examined. When the effects of magnesium compounds were examined separately, the Mg^{2+} concentration was adjusted to 0.9 mM for each compound. When the effects of sulfates were examined, the sulfate concentration was also adjusted to 0.9 mM for each compound.

Table S4. Categorization of photographs used for determining the localization of *R. erythropolis* PR4 cells in a one-round culture protocol using $MgSO_4$ for controlling the spatial arrangement of PR4 cells.

Conditions	Actions	Final Mg^{2+} concentrations	Cell localizations				n=
			Adhesion	ADH > TRN	TRN > ADH	Translocation	
Step 1	Addition of $MgSO_4$	0.9 µM	100	0	0	0	25
Step 2	Reduction of $MgSO_4$	0.09 µM	0	0	0	100	25
Step 3	Re-addition of $MgSO_4$	0.9 µM	100	0	0	0	25

Five independent experiments were performed, and 5 photographs were taken for each condition (n=25). The percentage of photographs categorized as ADH, TRN, ADH > TRN, or TRN > ADH was calculated for each condition examined.

Supplemental figure legends

Fig. S1. Typical localization of *Rhodococcus erythropolis* PR4 cells in alkane-containing two-phase culture. Panel A shows a representative photograph of translocated cells in the alkane phase (defined as translocation). Panel B shows adherent cells at the aqueous-alkane phase interface (defined as adhesion). These data were compiled from a previous report (4). Both photographs were taken at the same magnification. Bar = 2 μm.

Fig. S2. Effect of magnesium-containing compounds on the localization of *Rhodococcus erythropolis* PR4 cells in two-phase culture based on NP medium containing C12 alkane. The Mg^{2+} concentration was adjusted as 0.9 μM for each compound. A, $Mg(CH_3COO)_2$; B, $Mg(NO_3)_2$; C, $Mg(CH_3\text{-}CH[OH]\text{-}COO)_2$; D, $MgSO_4$. All photographs were taken at the same magnification. Bar = 10 μm.

Fig. S3. Effect of adding magnesium-containing compounds during cultivation on the inhibition of translocation to the C12 phase in the two-phase culture. A, $Mg(CH_3COO)_2$; B, $Mg(NO_3)_2$; C, $Mg(CH_3\text{-}CH[OH]\text{-}COO)_2$; D, $MgCl_2$. All photographs were taken at the same magnification. Bar = 10 μm.

Fig. S4. Release of cells from the aqueous-C12 interface to the aqueous phase induced by the addition of glucose and C8. Bar = 10 μm.

Fig. S1., Takihara *et al*.

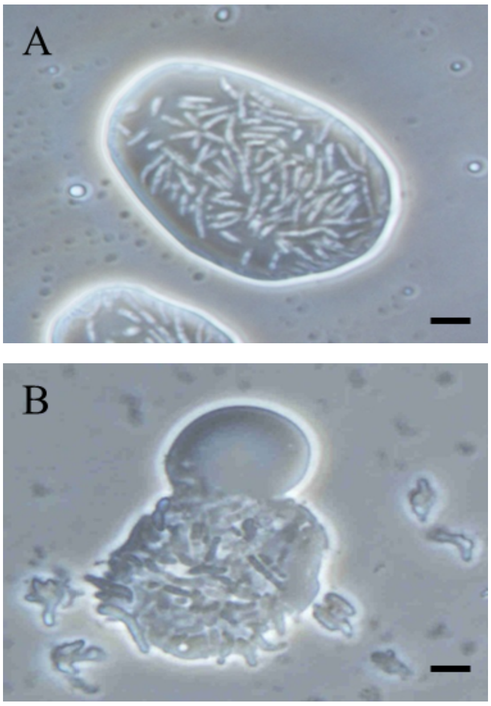

Fig. S2., Takihara *et al*.

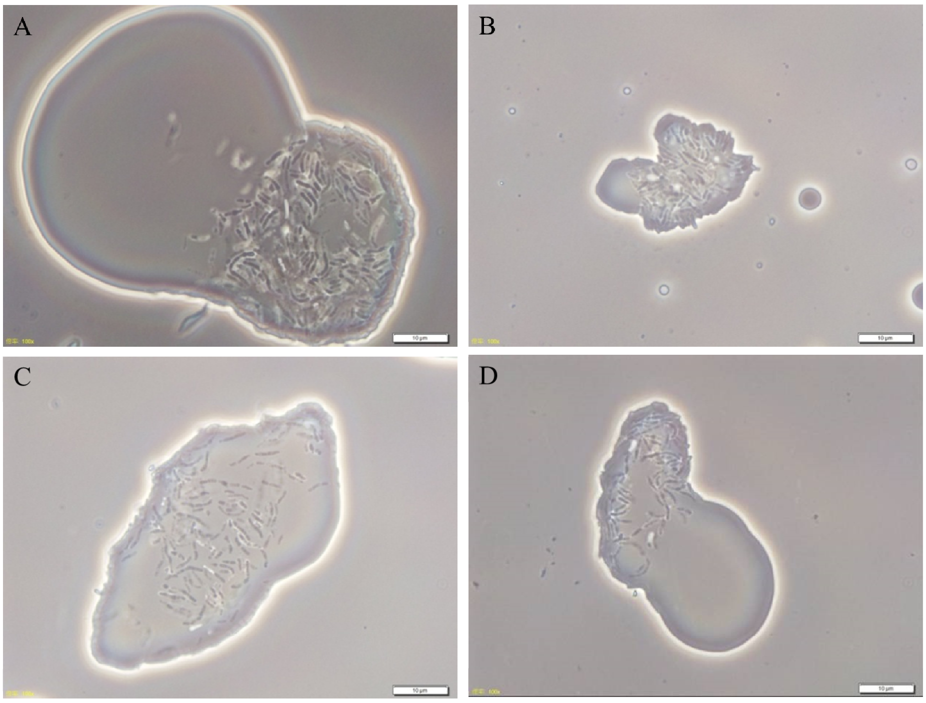

Fig. S3., Takihara *et al*.

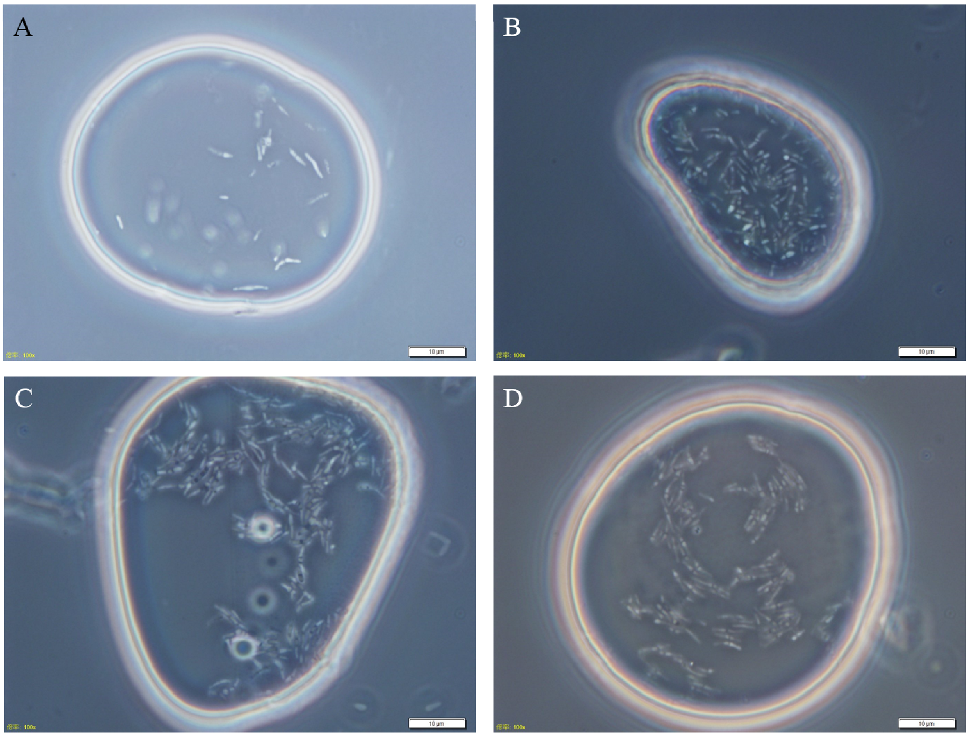

Fig. S4., Takihara *et al*.

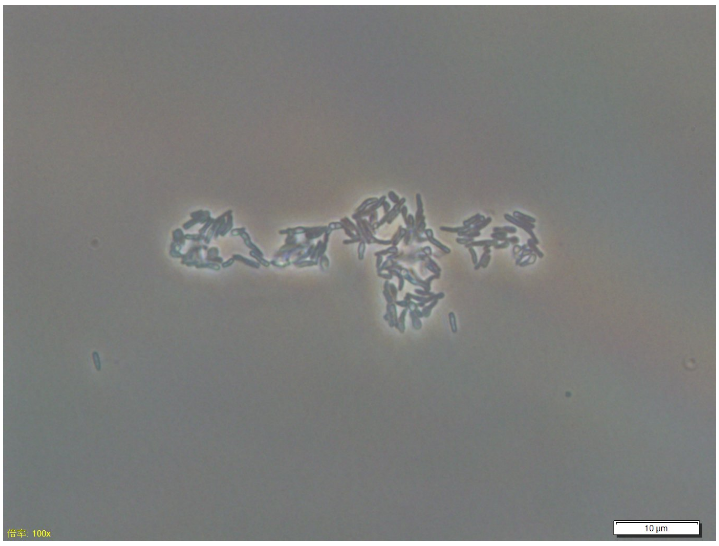

Supplement: Supplementary file 1 [file 31_178_s1.pdf]
